# Effective dose estimation for oncological and neurological PET/CT procedures

**DOI:** 10.1186/s13550-017-0272-5

**Published:** 2017-04-24

**Authors:** Josep M. Martí-Climent, Elena Prieto, Verónica Morán, Lidia Sancho, Macarena Rodríguez-Fraile, Javier Arbizu, María J. García-Velloso, José A. Richter

**Affiliations:** 1 Nuclear Medicine Department, Clínica Universidad de Navarra, 36, Pío XII Avenue, 31008 Pamplona, Spain; 2 IdisNA, Instituto de Investigación Sanitaria de Navarra, Pamplona, Spain

**Keywords:** PET/CT, Effective dose, Radiopharmaceutical, Whole body, Brain

## Abstract

**Background:**

The aim of this study was to retrospectively evaluate the patient effective dose (ED) for different PET/CT procedures performed with a variety of PET radiopharmaceutical compounds.

PET/CT studies of 210 patients were reviewed including Torso (*n* = 123), Whole body (WB) (*n* = 36), Head and Neck Tumor (HNT) (*n* = 10), and Brain (*n* = 41) protocols with ^18^FDG (*n* = 170), ^11^C-CHOL (*n* = 10), ^18^FDOPA (*n* = 10), ^11^C-MET (*n* = 10), and ^18^F-florbetapir (*n* = 10). ED was calculated using conversion factors applied to the radiotracer activity and to the CT dose-length product.

**Results:**

Total ED (mean ± SD) for Torso-^11^C-CHOL, Torso-^18^FDG, WB-^18^FDG, and HNT-^18^FDG protocols were 13.5 ± 2.2, 16.5 ± 4.5, 20.0 ± 5.6, and 15.4 ± 2.8 mSv, respectively, where CT represented 77, 62, 69, and 63% of the protocol ED, respectively. For ^18^FDG, ^18^FDOPA, ^11^C-MET, and ^18^F-florbetapir brain PET/CT studies, ED values (mean ± SD) were 6.4 ± 0.6, 4.6 ± 0.4, 5.2 ± 0.5, and 9.1 ± 0.4 mSv, respectively, and the corresponding CT contributions were 11, 14, 23, and 26%, respectively. In ^18^FDG PET/CT, variations in scan length and arm position produced significant differences in CT ED (*p* < 0.01). For dual-time-point imaging, the CT ED (mean ± SD) for the delayed scan was 3.8 ± 1.5 mSv.

**Conclusions:**

The mean ED for body and brain PET/CT protocols with different radiopharmaceuticals ranged between 4.6 and 20.0 mSv. The major contributor to total ED for body protocols is CT, whereas for brain studies, it is the PET radiopharmaceutical.

## Background

PET/CT is a dual-modality technique combining positron emission tomography (PET) with x-ray computed tomography (CT) [1]. Wide ranges of radiopharmaceuticals are used for studying different pathologies.

The most common isotope is ^18^F as a label for the 2-[^18^F]fluoro-2-deoxy-D-glucose molecule (^18^FDG) [2]. Currently, PET/CT with ^18^FDG is a common diagnostic tool in oncology for staging and treatment evaluation [3, 4]. Thus, tumor imaging with ^18^FDG PET has been identified in the project “Study on European Population Doses from Medical Exposure” (DDM2) as the fourth procedure among the highest contributors to the collective effective dose in all DDM2 countries, producing a median contribution of 16.2% to total per capita effective dose in nuclear medicine procedures [5]. A PET scan has an average frequency of 0.8 per 1000 of population for European countries, and more than half the countries reported that the use of PET/CT for oncological imaging has increased. The reported administered activity for the different countries ranged from 240 to 433 MBq. The average effective dose of PET for tumor imaging was 6.7 mSv, with a wide range of variation (the ratio between maximum and minimum values was 1.7). Although the CT patient dose in PET/CT was not reported, on average, 32% of CT scanners were used as diagnostic studies. However, the centers that participated in the DDM2 study showed considerable variation from country to country: in France, CT scanners in hybrid systems were used only for attenuation correction, whereas, in Italy, they were used for diagnostic purposes. No data was included in the DDM2 study regarding brain PET/CT studies and PET tracers other than FDG [6], and dosimetric studies for those protocols are scarce.

For “whole body” FDG PET/CT procedures, the relative contribution of the PET and CT effective dose has been reported to vary, with the CT component contributing between 54 and 81% of the total combined dose, depending on the CT parameters with the same scan length [7]. Furthermore, the term whole body protocol has been used to cover different body extensions: from vertex to mid-thighs [8], base of scull to upper thighs [7], and head to feet [9].

Dual-time-point imaging is a useful procedure for differentiating inflammatory and malignant processes and has been found to enhance the specificity of ^18^FDG PET imaging for diagnostic and prognostic purposes [10], with applications for both body [11] and brain protocols [12]. The dosimetric impact of the second PET/CT scan is produced by the CT part of the protocol.

In order to estimate patient dose in PET/CT and to establish diagnostic reference levels (DRL), it is important to collect data on administered activity of PET tracer and dose-length product (DLP) or volume computed tomography dose index (CTDI_vol_) in CT. These parameters are established as a DRL in some countries [13]. The effective dose can then be calculated. Effective dose is defined by the International Commission on Radiological Protection as the tissue-weighted sum of the equivalent doses in all specified tissues and organs of the human body and represents the stochastic health risk to the whole body.

The purpose of this study was to estimate patient effective dose of PET/CT procedures with the final motivation of patient dose optimization. To this end, DRL-related parameters were evaluated for different PET/CT procedures performed using a variety of PET radiopharmaceutical compounds, including oncological (covering different scan lengths) and neurological scans. The dosimetric impact of scan length, arm position, and dual time point imaging were studied. This dose evaluation is the initial step to subsequently achieving patient dose optimization.

## Methods

PET/CT studies were performed with a Biograph mCT TrueV scanner (Siemens Healthcare, Knoxville, TN, USA), which has a PET that covers a 21.8-cm axial field of view and a 64-detector CT scanner [14]. PET scan is performed in stop-and-go imaging mode, acquiring information in a variable number of bed positions along the patient. Each bed has an overlap of 9.5 cm with the contiguous bed.

For each patient, data recorded included diagnostic protocol, patient demographic data (sex, age, weight, and height), CT exposure parameters (tube voltage (kV), effective tube current-time product (mAs), rotation time, pitch, slice collimation, CTDI_vol_, and DLP), administered radiopharmaceutical and its activity, and PET scan parameters (number of bed positions). The automatic exposure control (“CARE Dose” package by Siemens) was used in all exams to acquire CT scans according to the lowest possible patient dose, based on the reference tube current-time product (reference mAs) defined by the user. When a dual-time imaging protocol was prescribed, the CT exposure parameters were also recorded for the second PET/CT study.

The PET/CT protocols, with ^18^FDG, [methyl-^11^C]-choline (^11^C-CHOL), l-[methyl-^11^C]-methionine (^11^C-MET), [^18^F]fluoro-l-dopa (^18^FDOPA), or ^18^F-florbetapir [15], were as follows:

- Torso imaging (Torso) covering from the base of the skull to mid-thigh (usually with arms above the head)
- Whole body (WB) examination from the top of the head to the feet, performed in two steps: the first step covered the head and torso (H&Torso) followed by PET/CT from the middle of the pelvis to the toes (Limbs)
- Head and neck tumor (HNT) protocol performed in two steps, the head and neck portion (H&N) with the arms down, and a scan from the apex of the lung to the mid-thigh (Trunk) with the arms up
- Brain examinations covering the head within one bed position

Patient data were retrospectively collected for a month from hospital files and PACS. For some infrequent brain PET/CT studies (^18^FDOPA, ^11^C-MET, and ^18^F-florbetapir), the registration period had to be extended to obtain a sample of 10. Finally, studies of 210 patients were reviewed, including 170 PETs with ^18^FDG, 10 with ^11^C-CHOL, 10 with ^18^FDOPA, 10 with ^11^C-MET, and 10 with ^18^F-florbetapir. This study has been approved by the institutional review board (Comité Ético de Investigación Clínica de Navarra). The need for informed consent was waived, and data anonymization provided the privacy guarantee.

For each patient, effective dose was evaluated using standard coefficients referring to a generic reference individual. Thus, effective dose (ED) from the CT exam was calculated using the conversion factors *k* (mSv/mGy cm) multiplied by the DLP [16], depending on the scanned region (Table 1). The PET ED was calculated by multiplying the injected activity by the *Γ* dose coefficient for each radiopharmaceutical. The *Γ* coefficients in mSv/MBq were 0.019 for ^18^FDG [17], 0.0084 for ^11^C-methionine [17], 0.025 for ^18^FDOPA [17], 0.0044 for ^11^C-choline [18], and 0.0186 for ^11^F-florbetapir [19]. The total ED associated with the combined PET/CT exam was evaluated as the sum of the PET and CT ED values.

**Table 1:** Dose conversion factors from DLP to ED

CT Scan in PET/CT protocol	Anatomical area	*k* = ED/DLP (mSv/mGy cm)	Reference
Brain	Head	0.0024	[34]
H&N	Head/neck	0.0090	[34]
Dual time	Chest	0.0204	[34]
Dual time	Abdomen	0.0163	[34]
Dual time	Pelvis	0.0143	[34]
Dual time	Abdomen/pelvis	0.0171	[34]
Trunk, Torso	Chest/abdomen/pelvis	0.0186	[34]
H&Torso	Whole body	0.0154	[34]
Limbs	Lower extremities	0.006 male	[35]
		0.0073 female	

Statistical analysis was performed using SPSS v20 (IBM Corporation). Results are expressed as mean ± standard deviation (minimum, maximum). Statistical differences between groups were evaluated using parametric or non-parametric tests depending on the normality of the data, and they were considered significant for *p* < 0.05.

## Results

The main technical CT parameters used in each protocol are shown in Table 2. Tube voltage was set to 120 kV, and collimation was fixed to 16 × 1.2 mm. The same parameters were used for the Torso, Trunk, H&Torso, H&N and Limbs protocols. CT parameters were adapted to each brain protocol to provide different image quality with different diagnostic value.

**Table 2:** Main technical parameters used for the CT protocols

Diagnostic protocol	CT part	Rotation time (s)	Pitch	Reference current (mAs)
Torso-^11^C-CHOL	Torso	0.5	1	120
Torso-^18^FDG	Torso	0.5	1	120
WB-^18^FDG	H&Torso	0.5	1	120
	Limbs	0.5	1	120
HNT-^18^FDG	Trunk	0.5	1	120
	H&N	0.5	1	120
Brain-^18^FDG	Brain	1	0.55	120
Brain-^18^FDOPA	Brain	1	0.55	100
Brain-^11^C-MET	Brain	1	0.8	200
Brain-^18^F-florbetapir	Brain	1	0.55	400

A total of 210 adult patients (130 men) were included in the study. Patient demographic characteristics (mean ± SD) were age, 61 ± 13 (21, 95) years old; weight, 73 ± 14 (40, 125) kg; and height, 167 ± 9 (140, 190) cm. The number of patients per diagnostic procedure with the corresponding PET scan parameters (administered activity and number of acquired bed positions) and CT exposure parameters (effective tube current, CTDI_vol,_ and DLP) is shown in Table 3.

**Table 3:** PET scan parameters and CT exposure parameters for each protocol

Diagnostic protocol	No. of patients	Activity (MBq)	Number of bed positions Median, %	CT part	Effective tube current-time product (mAs)	CTDI_vol_ (mGy)	DLP (mGy cm)
Torso-^11^C-CHOL	10	695 ± 44	6, 80%	Torso	89 ± 17 (57, 113)	6.5 ± 1.3 (4.2, 8.3)	562 ± 120 (317, 729)
Torso-^18^FDG	113	323 ± 63	6, 66%	Torso	87 ± 27 (35, 227)	6.3 ± 1.9 (3.8, 17.0)	557 ± 208 (287, 1876)
WB-^18^FDG	36	321 ± 71	7, 44%	H&Torso	109 ± 35 (55, 192)	7.9 ± 2.6 (4.0, 14.0)	776 ± 294 (336, 1049)
			7, 53%	Limbs	44 ± 14 (26, 94)	3.3 ± 1.0 (1.9, 6.9)	307 ± 104 (170, 693)
HNT-^18^FDG	10	299 ± 43	5, 70%	Trunk	74 ± 16 (54, 106)	5.4 ± 1.2 (3.9, 7.8)	429 ± 109 (300, 684)
			2, 100%	H&N	67 ± 14 (44, 46)	4.9 ± 1.0 (3.2, 6.3)	187 ± 38 (123, 237)
Brain-^18^FDG	11	296 ± 33	1, 100%	Brain	84 ± 7 (74, 96)	13.1 ± 1.0 (11.6, 15.0)	302 ± 30 (247, 349)
Brain-^18^FDOPA	10	159 ± 19	1, 100%	Brain	72 ± 5 (65, 80)	11.6 ± 1.4 (10.2, 15.0)	271 ± 32 (237, 349)
Brain-^11^C-MET	10	453 ± 96	1, 100%	Brain	137 ± 12 (115, 155)	20.4 ± 1.9 (17.9, 24.1)	508 ± 45 (419, 564)
Brain-^18^F-florbetapir	10	378 ± 13	1, 100%	Brain	268 ± 25 (239, 321)	39.0 ± 3.8 (37.3, 50.0)	973 ± 89 (870, 1167)

Table 4 summarizes the effective dose produced by the different PET/CT diagnostic protocols. Total mean ED for the different protocols ranged from 4.6 to 20.0 mSv. For Torso, WB, and HNT protocols, effective doses for CT were greater than for PET, whereas for brain protocols, the PET study produced a higher ED than the CT component. CT ED for Torso-^11^C-CHOL, Torso-^18^FDG, WB-^18^FDG, and HNT-^18^FDG diagnostic protocols accounted for 77, 62, 69, and 63% of the total protocol ED, respectively, and the mean PET ED was 3.1 mSv for the^11^C-CHOL, ranging between 5.7 and 6.1 mSv for the ^18^FDG protocols. The most common exploration (oncological Torso-^18^FDG) produced 6.1 mSv due to PET radiotracer and 10.3 mSv due to CT.

**Table 4:** Effective dose for PET/CT protocols with PET and CT contributions*

Diagnostic protocol	PET ED (mSv)	CT part	CT ED (mSv)	Total ED (mSv)
Torso-^11^C-CHOL	3.1 ± 0.2 (2.8, 3.3)	Torso	10.4 ± 2.2 (5.9, 13.6)	13.5 ± 2.2 (9.2, 16.6)
Torso-^18^FDG	6.1 ± 1.2 (3.5, 9.5)	Torso	10.3 ± 3.8 (5.3, 34.9)	16.5 ± 4.5 (9.7, 42.1)
WB-^18^FDG	6.1 ± 1.3 (4.6, 10.6)	H&Torso	11.9 ± 4.5 (5.2, 21.7)	20.0 ± 5.6 (1.8, 32.7)
		Limbs	2.0 ± 0.8 (1.1, 5.1)	
HNT-^18^FDG	5.7 ± 0.8 (4.7, 7.3)	Trunk	8.0 ± 2.0 (5.6, 12.7)	15.4 ± 2.8 (12.3, 22.1)
		H&N	1.7 ± 0.3 (1.1, 2.1)	
Brain-^18^FDG	5.6 ± 0.6 (4.3, 6.5)	Brain	0.73 ± 0.07 (0.59, 0.84)	6.4 ± 0.6 (5.0, 7.2)
Brain-^18^FDOPA	4.0 ± 0.5 (3.1, 4.5)	Brain	0.65 ± 0.08 (0.57, 0.84)	4.6 ± 0.4 (3.9, 5.2)
Brain-^11^C-MET	4.0 ± 0.8 (2.9, 5.2)	Brain	1.22 ± 0.1 (1.0, 1.4)	5.2 ± 0.5 (4.1, 6.5)
Brain-^18^F-florbetapir	7.0 ± 0.2 (6.6, 7.4)	Brain	2.33 ± 0.2 (2.1, 2.8)	9.1 ± 0.4 (8.8, 10.2)

*Values are presented as mean ± SD (minimum, maximum)

CT ED in brain protocols was statistically different (Kruskal-Wallis test, *p* < 0.01); thus, for ^18^F-florbetapir studies, it was 3.6 times higher than for Brain-^18^FDOPA studies. CT effective dose was 11, 14, 23, and 26% of the total dose received by the patients in ^18^FDG, ^18^FDOPA, ^11^C-MET, and ^18^F-florbetapir brain PET/CT studies, respectively. PET ED was also statistically different among radiotracers (ANOVA *p* < 0.001). In particular, ^18^FDOPA and ^11^C-MET PET scans provided lower doses than ^18^FDG and ^18^F-florbetapir PET scans (Posthoc-Bonferroni test, alpha = 0.05, *p* < 0.01).

When arm position was considered for the Torso protocol, statistically significant differences (Mann-Whitney *U* test, *p* < 0.01) were found in mAs, CTDI_vol_, DLP, and CT ED between patients with arms above the head and patients with arms alongside the body. For these groups, the ED due to the CT examination (mean ± SD) was 9.7 ± 6.0 (5.3, 18.0) mSv and 17.1 ± 7.8 (8.0, 34.9) mSv, respectively.

In ^18^FDG PET/CT scans with different scan length (HNT, Torso, and WB protocols), no significant differences in radiotracer activity and PET effective dose were found, but the CT effective dose for the CT portion that included torso or trunk was statistically different (Kruskal-Wallis test, *p* < 0.01). Although CT parameters were the same, scan length was different. Thus, the CT effective dose increased according to the scanned length, with 8.0, 10.4, and 11.9 mSv for the trunk, torso, and H&Torso parts of the HNT, Torso, and WB protocols, with mean scanned patient length of 761, 839, and 926 mm, respectively.

Dual time PET/CT was performed in 32 patients. Only one corresponded to a brain-^18^FDG protocol, and in 31 patients (18% of the 169 body studies), a delayed PET/CT was performed for different parts of the torso. The frequency of the number of bed positions explored was 25, 66, and 9% for one, two, and three bed positions, respectively. The CT ED (mean ± SD) for the delayed imaging was 3.8 ± 1.5 (0.8, 6.9) mSv.

## Discussion

In this study, we evaluated DRL-related parameters and estimated patient effective dose for different PET/CT procedures performed using a variety of PET radiopharmaceutical compounds, including oncological ^18^FDG scans (covering different patient lengths) and brain studies.

This is the first ^18^FDG PET/CT study to evaluate the patient effective dose considering three different scanning lengths: torso scan (from base of the skull to mid-thigh), WB scan (from top of the head to the feet), and HNT examinations (from the vertex to the mid-thigh in two steps or scans). This analysis with three different scan lengths may provide an understanding of the differences among previously published results of PET/CT ED where different scan lengths have been considered (Table 5). Only Avramova-Cholakova et al. [9] have reported the impact of including limbs in ED. With regard to CT, in this study, the objective of the CT study was anatomical localization in most cases; the CT exposure parameters were fixed for each protocol, and no contrast enhancement was used in any case. However, as different protocols covered different patient lengths, a variety of conditions have been evaluated. Previously published studies have analyzed differences between adapted CT protocols for normal and obese patients or between contrast-enhanced CT [20] and CT adapted for high-quality or interspaced high-speed CT [21]. Others demonstrated an increase in CT dose when moving from a standard protocol to a diagnostic protocol [22] or from low-dose protocols to contrast-enhanced protocols [23]. Willowson et al. [8] showed that PET ED decreases from 6.3 to 6.0 mSv when ED is scaled to individual patient weight.

**Table 5:** Summary of published data on PET/CT parameters and patient effective dose for ^18^FDG whole body oncological studies

Reference	Scan length as per this study	Study from	Study to	Comment or CT description	Tube current mAs or mA and s	AEC	CTDI_vol_ (mGy)	DLP (mGy cm)	ED_CT (mSv)	Activity (MBq)	ED_PET (mSv)
[7]	Torso	Base of the skull	Upper-thigh	Impact of AEC	100–300 mA and 0.5 s	Yes	–	–	7.2	370	6.23
					250 mA and 0.5 s	No			18.6		
[36]	Torso	Base of the skull	Upper-thigh	TAI patients	112 mAs	Yes	–	–	14.4 ± 2.8	312 ± 43	4.4 ± 0.6
[22]	Torso	Base of the skull	Mid-thighs	Standard CT	39 mAs	Yes	–	438 ± 85	5.0 ± 1.0	450 ± 32	9.0 ± 1.6
				Diagnostic CT	120 mAs			1050 ± 342	15.4 ± 5.0		
[30]	H&Torso	Head	Mid-thighs	Initial CT	–	Yes	6.4 ± 2.4	537 ± 222	8.1 ± 3.3	–	–
				Optimized CT			4.3 ± 1.6	368 ± 145	5.5 ± 2.1		
[20]	H&Torso	Top of the skull	Mid-thighs	Most frequent CT	110 mA and 0.8 s	–	4.3	434	7.5	336 ± 49	5.6
				Special CT	250 mA and 0.5 s		6.0	616	10.95		
				Special CT	300 mA and 0.5 s		7.2	732	13.2		
[8]	H&Torso	Vertex	Mid-thighs	–	80 mAs	Yes	–	–	8.2	304 ± 45	6.3 ± 1.2
[21]	H&Torso	Head	Pelvis	High-quality CT	80 mA and 0.8 s	–	–	–	19.0	–	–
				High-speed CT					8.8		
[9]	H&Torso	Head	Mid-thighs	Center 1	84 mA and 0.5 s	Yes	5.9 ± 1	591 ± 130	7. 8	258 ± 56	4.9
				Center 2	–	Yes	5.4 ± 2	575 ± 248	7.7	313 ± 73	5.9
	WB	Head	Feet	Center 1	89 mA & 0.5 s	Yes	6.2 ± 1.4	1112 ± 285	9.4	258 ± 56	4.9
				Center 2	–	Yes	3.7 ± 1	620 ± 166	5.9	313 ± 73	5.9
[23]	–	–	Symphysis	Low-dose CT	–	–	1–2.9	–	1.3–4.5	300–370	5.7–7
				Contrast-enhanced CT			9.5–14.1	–	14.1–18.6		
This study	Torso	Base of the skull	Mid-thighs	–	120 mAs	Yes	6.3	557	10.3	323	6.1 ± 1.2
	HNT	Vertex	Mid-thighs	–			4.9 + 5.4	187 + 429	9.7 ± 2.2	299 ± 43	5.7 ± 0.8
	WB	Head	Feet	–			7.9 + 3.3	776 + 307	14.0 ± 4.9	321 ± 71	6.1 ± 1.3

CTDI_vol_ 75^th^ percentile of whole-body oncological PET/CT protocols submitted to the Society of Nuclear Medicine and Molecular Imaging Clinical Trials Network ranged from 9.7 to 10.2 mGy [24]. The wide ranges of CT acquisition parameters suggested that CTDI_vol_ reference levels may help to optimize CT protocols. In a French national survey for whole-body PET/CT [25], the average ^18^FDG administered activity, CTDI_vol_, and DLP were 310 MBq, 6.6 mGy, and 628 mGy cm, respectively. The ED whole-body CT (from neck to thighs) was 8.6 mSv. A Japanese national survey [26] also observed the different definition of whole-body protocol; the most commonly used extension was from the top of the head to the thigh. CT and ^18^FDG PET ED for male (female) patients were 10.1 (9.7) and 4.5 (3.7) mSv, respectively.

For ^18^FDG PET tumor imaging in our patient cohort, the mean PET effective dose, directly proportional to the administered activity, was approximately 6 mSv, within the range of the published data 4.9 to 9 mSv (from 258 to 450 MBq of ^18^FDG). In the European Union, only seven countries have DRL, ranging from 200 to 400 MBq [13], with a variability of 100% in the DRL and of 43% in activity (Table 5).

The CT ED depends on the scan operating parameters, scanning length, and patient area. For ED evaluation, this dependence is reflected in CTDI_vol_ and DLP parameters as well as in the dose conversion factor. Of the evaluated protocols, the H&Torso part of the WB protocol had the highest mean DLP value, and the *k* factor was slightly lower than the value for Trunk and Torso scans. The Trunk CT part of the HNT-^18^FDG protocol also had a lower DLP than the Torso CT scans. CT ED for Torso-^18^FDG and HNT-^18^FDG protocols were similar (10 mSv) and lower than for WB-^18^FDG scan (14 mSv). These values, which demonstrate dose dependence on the PET/CT protocol used, lie within the range of published data considering the PET/CT protocol (Table 5). For the WB protocol, ED may be potentially reduced if instead of performing two scans (one for H&Torso and another one for Limbs) with an overlap zone, a single CT study (without overlaping) would be performed.

The impact on the CT effective dose due to the position of the arms in a PET/CT study and the impact of the second PET/CT in a dual-time-point imaging scan have never been reported. In this study, for the Torso-^18^FDG protocol, we showed that ED increases by approximately a factor of two when the arms are alongside the body, compared to when the arms are positioned above the head. Additionally, for dual-time-point imaging, total ED was increased by 3.8 mSv in the 18% of the patients with body protocols.

Few references exist on patient dosimetry in brain studies, other than those reporting radiopharmaceutical biodistribution and organ dosimetry. This is probably because these protocols are less frequent and produce a lower dose in comparison with oncological ^18^FDG studies. In 2014, only Ireland had a DRL for ^18^FDG brain studies (290 MBq) [13].

Patient dosimetry for neurological PET/CT protocols with ^18^FDOPA, ^18^FDG, ^11^C-MET, and ^18^F-florbetapir have been evaluated. The purpose of the CT scan was different in each protocol, and the reference tube current was adjusted according to the required image quality for that specific purpose (e.g., anatomical localization, or diagnosis). In particular, the tube currents for the ^11^C-MET and ^18^F-florbetapir protocols were adjusted for diagnostic purposes. Hence, the mean CT ED in a Brain-^18^F-florbetapir study (2.33 mSv) was 3.6 times higher than the CT ED in a Brain-^18^FDOPA study (0.65 mSv). Thus, for amyloid brain imaging, it would be desirable to distinguish the relative accumulation of radiotracer binding between gray and white matter, as well as possible pitfalls due to bone deposits and atrophy. CT is also important in neurooncological studies using ^11^C-MET PET/CT studies, where the CT can help by adding structural information. CT doses reported in this study are similar to those in the study by Kaushik et al. [20], where brain CT parameters were adapted for obese patients and contrast-enhanced studies.

Effective doses due to the radiopharmaceutical compounds in the brain PET/CT protocols have been found to contribute between 77 and 88% of the total dose. Kaushik et al. evaluated a PET effective dose of 3.5 and 5.8 mSv for ^18^FDOPA and ^18^FDG brain PET/CT studies [20], respectively, similar to that found in this study. The PET dose for ^18^F-florbetapir corresponds to the dose reported for 370 MBq activity administration to a 70-kg patient [19].

Finally, further studies are needed in the field of patient dose optimization for each PET/CT protocol. The effective dose due to the PET scan is directly proportional to the administered dose. Hence, reducing the injected activity will provide a direct reduction in the patient dose. In addition, there will be a positive impact on the operating costs of the PET/CT facility and in the medical staff dosimetry performing the examinations. The new generation of PET scanners, which incorporate improved reconstruction methods with point spread function and time of flight information [27] and image-noise modeling [28], should be used to reduce the injected activity in the patient [29]. CT protocol optimization results in a reduction in the mean CT radiation dose and analysis of the image quality can show no clinically relevant degradation of the low-dose studies [30]. Typically, PET/CT tomographs acquire the PET scan in a stop-and-go mode, imaging a fixed number of bed positions with overlapping. Hence, the total length is usually more extense than the area of interest. In PET acquisition with a continuous table motion [31], the total scan length is not forced to be a specific number of bed positions and can be adjusted to any area of interest to be explored, hence reducing total CT scanned length and patient dose. Recent advances in CT such as iterative reconstruction [32] and adaptive kV [33] have shown a reduction in patient CT dose, additional to the dose reduction achieved with the tube current modulation used in this study.

The main limitation of this study is the simplified approach used to estimate effective dose, using the same conversion coefficient independently of weight, age, or sex of the patient. Therefore, the estimated doses refer to a generic reference individual. For a more realistic analysis of the actual PET doses received by each patient, additional information about the individual biokinetics and anatomical and physiological properties are required. However, this simple approach is suitable for the aim of this study, which was to estimate and compare population doses for different PET/CT procedures; this approach is also used elsewhere to obtain population PET and CT doses [8]. Another limitation is that all PET coefficients were obtained from the ICRP, except for those not included in the ICPR documents. Those coefficients were obtained from other publications on individual biokinetics and dosimetric studies, where the procedure for calculating the dose may not be fully equivalent to the one used by ICRP.

## Conclusions

The mean effective dose for body and brain PET/CT protocols with different radiopharmaceuticals (^18^F-FDG, ^11^C-choline, ^11^C-methionine, ^18^FDOPA, and ^18^F-florbetapir) ranged between 4.6 and 20.0 mSv. In body protocols, CT is the major contributor to the total effective dose. Moreover, the increase in total scan length, the position of the arms alongside the body instead of above the head, and the delayed PET/CT study in a dual-time protocol significantly increased the contribution of the CT effective dose to the total effective dose. Hence, inclusion of the head and legs in a body protocol, the positioning of the arms alongside the body, and the acquisition of delayed images should be justified for each patient in order to optimize the PET/CT protocol dose and to maintain the dose as low as reasonably achievable without compromising diagnostic purpose. For brain protocols, although the main contribution to the total effective dose was due to the PET radiopharmaceutical, CT can increase the dose by a factor of about 4 if it is used for diagnostic purposes.

## Abbreviations

^11^C-CHOL: Methyl-[^11^C]-choline
^11^C-MET: l-[Methyl-^11^C]-methionine
^18^FDG: 2-[^18^F]Fluoro-2-deoxy-D-glucose molecule
^18^FDOPA: [^18^F]Fluoro-l-DOPA
CT: Computed tomography
CTDI_vol_: Volume computed tomography dose index
DLP: Dose-length product
DRL: Dose reference level
ED: Effective dose
H&Torso: Head and Torso
HNT: Head and Neck Tumor
PET: Positron-emission tomography
WB: Whole body
